# Neuralgias and Neuralgic Affections

**Published:** 1892-11

**Authors:** 


### Neuralgias and Neuralgic Affections.

At a recent meeting of the Medical College of Vienna, Benedikt read a paper on the above subject, in which he distinguished three kinds of neuralgias: (1) Of the nerve trunks or plexuses; (2) of the nerve roots; (3) of the terminations of the nerves.

In neuralgias of the nerve trunks and plexuses there are not only pains during the attacks, but apart from the attacks there are painful points over the tract of the nerve. In most cases all the nerves which issue from a plexus are more or less affected.

The prognosis of idiopathic affections of the nerve trunks and plexuses is very favorable when the specific treatment is applied from the first. Among the specific medicines, Benedikt mentions in terms of special approbation iodine and subcutaneous injections of phenic acid. The salicylates and antipyrine have a curative effect only when the natural duration of the affection is short. Narcotics should be given as little as possible, as they produce only a deceptive lull. The truly specific modes of treatment are galvanization and punctiform cauterizations. Benedikt compels the eschars following the cauterizations to suppurate for eight or ten days by means of an epispastic application, and never has recourse to more than one cauterization. This treatment is, in his estimation, so efficacious that when it fails one may affirm that there exists in the neighborhood of the nerve a lesion not yet appreciable, or a constitutional disease, and that this is the cause of the neuralgia.

Neuralgias of the nerve-roots are characterized by very intense intermittent pains without *points douloureux.* The nerve is painful to the touch, but this pain is alleviated by pressure. This variety of pain is met with in ataxia and in certain painful tics. These neuralgias at the commencement are unilateral; they denote often an alteration of the spinal meninges, in which case they do not coincide with neuralgias of the nerve trunks and plexuses—while the eccentric idiopathic neuralgias are often associated with peripheral pains or invade the nerve terminations. The unilateral, eccentric, idiopathic neuralgias of the roots have no tendency to follow an ascending course. Galvanization employed against these neuralgias gives no result, while faradization *loco dolenti* exercises a calmative but not curative action. On the other hand, the electric cauterizations over the seat of these neuralgias have a very favorable action. The cauterization must be applied over the roots which contain the sensory fibers of the region affected.

In the neuralgic affections of the nerve terminations (anthralgias, aphalagias) cauterization has also a very satisfactory effect. Faradization and the electro-static douche are very efficacious against migraine. As most patients can not stand the treatment during the attack, it is better to carry it out in the intervals of the pains, beginning with three *seances* per week, then two, then only one; but the treatment must be persevered with a long time, from nine months to a year in many cases.—*Boston. Med. and Surg. Jour.*

